# *SDF1* Gene Variation Is Associated with Circulating SDF1α Level and Endothelial Progenitor Cell Number–The Bruneck Study

**DOI:** 10.1371/journal.pone.0004061

**Published:** 2008-12-30

**Authors:** Qingzhong Xiao, Shu Ye, Friedrich Oberhollenzer, Agnes Mayr, Marjan Jahangiri, Johann Willeit, Stefan Kiechl, Qingbo Xu

**Affiliations:** 1 Cardiovascular Division, King's College London BHF Centre, London, United Kingdom; 2 Barts and the London School of Medicine and Dentistry, Queen Mary University of London, London, United Kingdom; 3 Department of Internal and Laboratory Medicine, Bruneck Hospital, Bruneck, Italy; 4 Department of Cardiothoracic Surgery, St. George's Hospital, London, United Kingdom; 5 Department of Neurology, Medical University of Innsbruck, Innsbruck, Austria; Leiden University Medical Center, Netherlands

## Abstract

**Background:**

Stromal cell-derived factor-1 (SDF1) and its receptor CXC chemokine receptor 4 (CXCR4) play a critical role in progenitor cell homing, mobilization and differentiation. It would be interesting to assess the predictive value of SDF-1alpha level for EPC number, and to ascertain whether there is a relationship between *SDF1* gene variation, plasma SDF-1alpha level, and the number and function of circulating EPCs. We also tested whether EPC number and function was related to CXCR4 gene variation.

**Methodology and Principal Findings:**

We genotyped a cohort of individuals who participated in the Bruneck Study for single nucleotide polymorphisms (SNPs) in the *SDF1* and *CXCR4* genes, and measured blood SDF1α level as well as EPC number and function. SDF1α levels were correlated with age, gender, alcohol consumption, circulating reticulocyte numbers, and concentrations of matrix metalloproteinase-9, C-reactive protein, cystatin C, fibrinogen and homocytein. In blood samples taken in 2005, EPC number was inversely associated with SDF1α level (p<0.001). EPC number in 2005 was also inversely associated with SDF1α level in 2000 (p = 0.009), suggesting a predictive value of plasma SDF1α level for EPC number. There was an association between the *SDF1* gene rs2297630 SNP A/A genotype, increased SDF1α level (p = 0.002) and lower EPC number (p = 0.006).

**Conclusions:**

Our data indicate that a SDF1 gene variation (rs2297630) has an influence on SDF1α level and circulating EPC number, and that plasma SDF1α level is a predictor of EPC number.

## Introduction

Asahara and colleagues in 1997 demonstrated for the first time that purified CD34 positive haematopoietic progenitor cells from peripheral blood could differentiate, ex-vivo, into an endothelial phenotype and were named Endothelial Progenitor Cells (EPCs)[1]. Growing evidence indicates that EPCs play a major role in angiogenesis and vasculogenesis[2], [3]. Indeed, mobilized EPCs can promote new blood vessel formation in ischaemic tissues, enhancing perfusion and recovery[2], [3]. Ex vivo expanded EPCs isolated from peripheral blood can also incorporate into the site of myocardial neovascularization[4], and intracoronary infusion of peripheral blood or bone marrow-derived progenitors in patients with acute myocardial infarction was shown to significantly enhance post-infarction remodelling[5], [6]. Moreover, EPC numbers have a prognostic value and can be used as a predictive biomarker in the cardiovascular diseases[7]–[9]. Other investigators have also shown reduced EPC number in patients with risk factors for cardiovascular diseases such as diabetes[10], [11] and smoking[12], with cessation of smoking resulting in a return of EPC numbers to normal. EPC number is also reduced in groups of patients known to be at higher risk of cardiovascular disease such as those with rheumatoid arthritis[13] or chronic renal failure[14]. EPC number is also known to be reduced in established non-coronary cardiovascular disorders such as in patients with strokes[15], peripheral vascular disease[10] and patients with erectile dysfunction[16]. However, our recent data from a population-based, longitudinal study refuted the traditional view that the EPC number is negatively related to cardiovascular risk factors. We showed that changes of EPC numbers are loosely associated with certain risk factors for the cardiovascular disease and not directly associate with the disease development[17].

Animal studies have indicated that SDF1 (also known as CXC chemokine ligans 12) and its receptor CXC chemokine receptor 4 (CXCR4) plays a critical role in progenitor cell homing, mobilization and differentiation. Inactivation of the *SDF1* or *CXCR4* gene in mice led to early embryonic lethality due to abnormality in the cerebellar and gastrointestinal vasculature and in hematopoiesis development[18]–[20]. The number of circulating hematopoietic stem cells (HSCs) or EPCs were increased by *SDF1* gene transfer using the adenovirus infection technique[21]–[23]. Overexpression of SDF1 in ischemic tissues has been found to enhance EPC recruitment from peripheral blood and to induce neoangiogenesis in ischemic tissues[24], [25]. Recent evidence also suggests that SDF1α is a driving force for EPC differentiation[26]. All these findings from animal studies strongly suggest that SDF1α has a crucial role in stem/progenitor cell mobilization, differentiation, and injured tissues-specific homing. However, it remained unknown whether SDF1α played such a role in humans. Therefore, we recently examined the levels of SDF1α and several angiogenic cytokines in relation to circulating EPC numbers in a population-based study. We found that plasma levels of SDF1α, but not VEGF or G-CSF, were strongly associated with EPC number and function, suggesting a role of SDF1α in EPC mobilization and differentiation in humans[17]. In that study, the association of SDF1α levels with EPC numbers was detected using blood samples taken at the same time point. In the present study, we investigated whether SDF1α levels had a long-term predictive value for EPC numbers.

There is emerging evidence indicating that variation in the human *SDF1* gene can have an influence on SDF1α levels[27], [28], which was demonstrated clearly to be involved in progenitor cell mobilization and differentiation in human and animal studies. Moreover, previous study showed that EPC number is, at least in part, genetically regulated[29], and the presence of SDF1-3'A allele was a predictive factor of CD34^+^ cell mobilization[30], which prompt us to hypothesize that the SDF-1 gene single-nucleotide polymorphism (SNP) might involve in their gene transcription and progenitor cell mobilization, differentiation and homing. In the present study, we examined whether there was a relationship between *SDF1* gene variation, SDF1α level and circulating EPC number. In addition, we tested whether there was also a relationship between variation in the gene encoding the SDF1α receptor CXCR4 and EPC number.

## Methods

### Study Population and clinical variables

The subjects of this study were residents of the Bruneck area in the Bolzano Province of Italy, who participated in the Bruneck Study[31]. Details of the Bruneck study have been described previously[31]. DNA samples were available for 826 subjects, and these were genotyped single nucleotide polymorphisms (SNPs) in the *SDF1* and *CXCR4* genes. Blood samples taken in 2000 (n = 684) and 2005 (n = 574) respectively were used for measurements of SDF1α levels. EPC number and function were assessed in blood samples collected in 2005 (n = 571 for EPC number and n = 542 for EPC colonies number respectively). Subjects with and those without EPC number and EPC function assessments did not differ in age, sex and cardiovascular risk factors. The appropriate ethics committees (Autonome Provinz Bozen-Sanitatsbetrieb Bozen Ethikkomittee) approved the study protocol and all study subjects gave their written informed consent before entering the study.

Systolic and diastolic blood pressure was taken with a standard mercury sphygmomanometer after at least 10 min of rest (mean of three independent measurements). Hypertension was defined as blood pressure ≥140/90 mm Hg or the use of anti-hypertensive drugs. The average number of cigarettes smoked per day was noted for each smoker and ex-smoker. Diabetes was diagnosed according to ADA criteria. Assessment of regular alcohol consumption was performed with a standardized questionnaireand quantified in terms of grams per day (g/day).

### EPC culture assay

EPC numbers in the blood samples were determined as described previously[17]. Briefly, positive stained cells for DiI-Ac-LDL and Lectin were considered to be EPCs on day 5 of culture. The total numbers of EPC per well were counted by two trained independent senior investigators blinded to the clinical details of the subjects. The EPC in a minimum of two wells were counted and the average was then recorded.

### EPC-colony formation unit (CFU) assay

The EPC-CFU assay performed as described previously[17]. Briefly, PBMNC were resuspended in EPC culture medium (M199 with 20%FCS and antibiotics) and then plated on fibronectin coated 6 well plates at a concentration of 5 million cells per well. The endothelial colonies were counted manually on day 7. Strict guidelines were followed to ensure consistent counting of EPC colonies. Two senior investigators who were blinded to the subjects' clinical status counted the colonies.

### Enzyme-linked immunosorbent assay (ELISA)

Commercially available SDF1α and matrix metalloproteinase-9 (MMP9) ELISA kits (Quantikine, R&D Systems, UK) were used to determine plasma SDF1α and MMP9 levels. All ELISA tests were carried out at room temperature on freshly thawed plasma samples. The concentration was determined by comparison with a standard curve, following manufacturer's instruction. Other laboratory parameters and vascular risk factors were all examined by standard methods[32].

### Genotype analyses

We selected tagging SNPs in the *SDF1* and *CXCR4* genes from the HapMap database (data release in June 2005). Three SNPs in *SDF1* (rs2297630, rs266087 and rs1413519) and two in *CXCR4* (rs16832740 and rs12691874) to capture SNPs with a minor allele frequency >0.2 at these loci and R^2^>0.8 were typed in this study. In addition, we studied the rs266085 SNP which had been reported to influence SDF1 expression[27] and the rs1801157 SNP which had suggested to be associated with plasma SDF1 level[28]. Genomic DNA was extracted from blood samples of the Bruneck study subjects, and genotypes were determined using the TaqMan genotyping method.

### Statistical Analysis

The data were analyzed using the SPSS 15.0 and BMDP software packages. Continuous variables were presented as means±SD or medians (interquartile range), and dichotomous variables as percentages. Correlations between EPC number and EPC-CFU, SDF1α level and other parameters were estimated by calculation of crude and partial (age/sex-corrected) Pearson correlation coefficients. Variables with a skewed distribution were logB_eB_-transformed. Non-parametric tests yielded very similar findings (data not presented). Associations of EPC number, EPC-CFU and SDF1α level with SDF1 and CXCR4 genotypes were assessed using generalized linear models adjusted for age and sex. Multivariate models additionally included standard cardiovascular risk factors and the factors associated with SDF1α levels (LDL and HDL cholesterol concentrations, smoking, hypertension, diabetes, alcohol consumption, and levels of CRP, MMP9, cystatin C, fibrinogen and homocystein). A Bonferroni correction was performed to account for the multiple comparisons (5 SNPs in SDF1 and 2 in CXCR4) performed.

## Results

### Relationship of plasma SDF1α level with inflammatory markers and other variables

**Figure 1:**
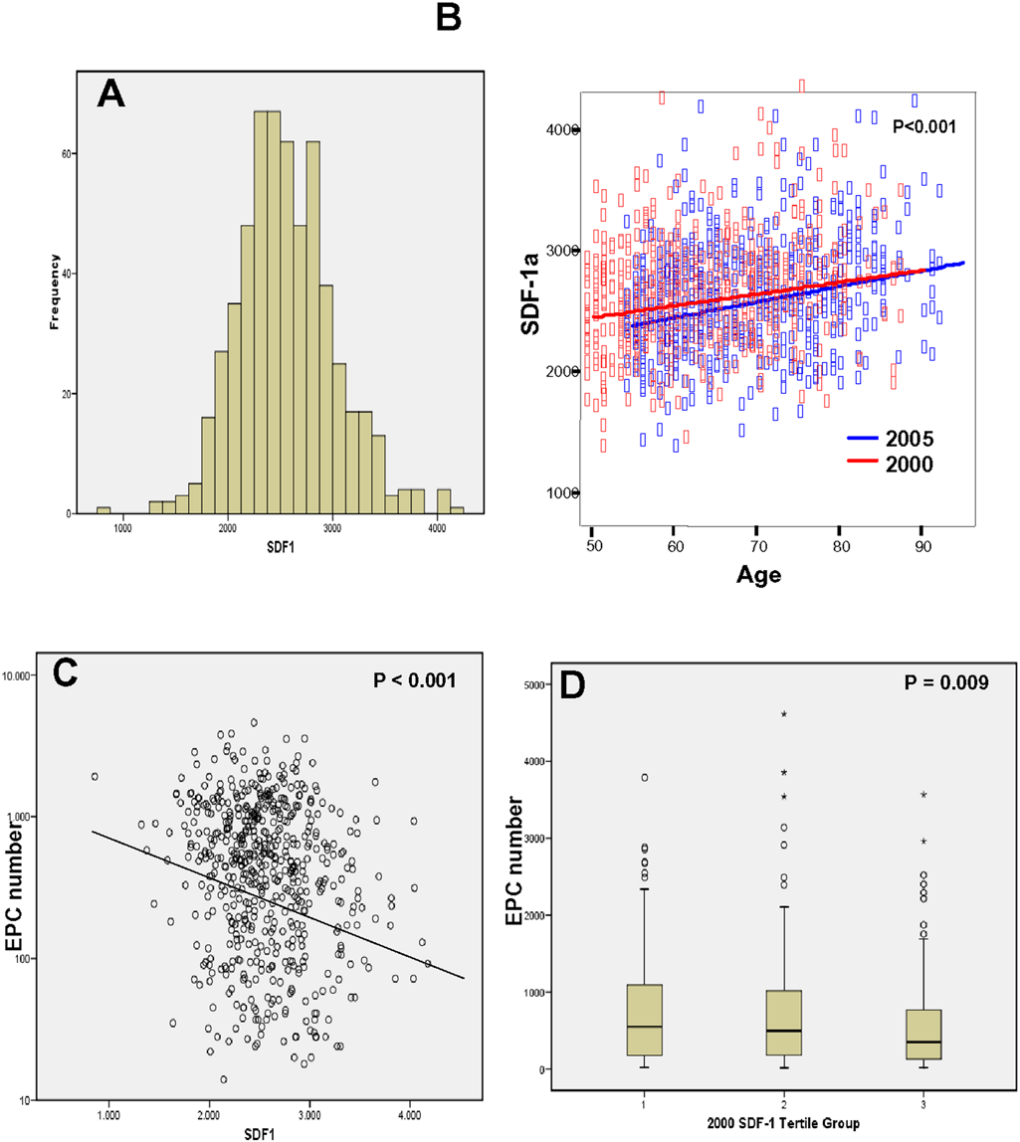
Panel A shows near normal distribution of SDF1α in the Bruncek study population. Panel B displays the association between SDF-1α measured in 2000 and in 2005 and age (r = 0.270, p<0.001). Panel C illustrates the correlation between SDF1α levels and EPC number in 2005. The regression line demonstrates clearly that SDF1α levels are inverse association with EPC number. Panel D illustrates the association between EPC numbers in 2005 and SDF1α level in 2000 (tertile groups). SDF-1 tertile groups are defined as follows: T1<2409, T2 2409–2753 and T3>2753. The box plots indicating EPC number median and IQRs. Notably, EPC numbers are significant associated inversely with SDF-1α levels, especially much low EPC number were likely observed in the top tertile group of SDF-1 levels.

Near normal distribution of SDF1α was observed in our study (**Figure 1A** **.** Kolmogorov-Smirnov p>0.05**)**. Mean SDF1α levels in 2000 and 2005 were 2652 pg/ml and 2565 pg/ml, with a range of 880 pg/ml to 5595 pg/ml and 1322 pg/ml to 4174 pg/ml, respectively. SDF1α levels in 2000 and 2005 were highly correlated (intraclass correlation* = *0.495; *P*<0.001), indicating that the SDF1α level is relatively consistent over time. As expected, both plasma SDF1α level measured in 2000 (r = 0.207, p<0.001) and 2005 (r = 0.270, p<0.001) increased with age (**Figure 1B**) and was higher in women (2619 in women vs. 2497 in men, p = 0.002). Moreover, plasma SDF1α level was correlated with alcohol consumption (age/sex-adjusted correlation coefficient r = −0.087, p = 0.042), reticulocyte numbers (r = −0.132, p = 0.002), plasma levels of matrix metalloproteinase-9 (MMP9) (r = 0.146, p = 0.001), C-reactive protein (hsCRP) (r = 0.090, p = 0.037), cystatin C (r = 0.277, p<0.001), fibrinogen (r = 0.159, p<0.001), and homocytein (r = 0.174, p = 0.001). (**Table 1**)

**Table 1:** Association between SDF-1α level and selected vascular risk factors and laboratory parameters (2005).

Variable	SDF-1α tertile group (pg/ml)			P value for trend
	Low Tertile (lower than 2350)	Medium Tertile (2350–2743)	High Tertile (higher than 2743)	
Alcohol consumption (g/day)	21.9±27.4	17.9±23.9	15.1±24.5	0.042
Reticulocytes (‰)	12.7±3.42	12.2±3.30	11.7±3.15	0.002
MMP9 (ng/mL)	69.2±60.6	80.5±59.0	87.3±60.2	0.001
High-sensitivity CRP (mg/L)	3.5±4.7	3.1±3.6	5.1±9.9	0.037
Fibrinogen (mg/dL)	285.4±53.8	296.4±54.2	311.4±64.2	0.009
Homocystein (µmol/L)	11.6±5.9	11.4±5.0	13.7±7.4	0.017
Cystatin C (mg/L)	0.92±0.16	0.98±0.23	1.12±0.31	<0.001

Values are means ±SD. P values for trend are from age- and sex-adjusted analyses.

### Plasma SDF1α level predicts EPC number

EPC number and EPC-CFU number (per 1 ml blood) displayed a non-normal distribution, with the majority of subjects (more than 75%) having 0 to 1000 EPC and 0 to 340 EPC-CFU, respectively. SDF1α levels were significantly and inversely associated with EPC number (p<0.001) in samples collected in 2005 (Figure **1C** **)**, indicating that SDF1α has an influence on EPC number in the general population. Importantly, EPC number in 2005 was also associated with SDF1α level in 2000 (p = 0.009), indicating a long-term predictive value of SDF1α level for circulating EPC number (**Figure 1D**). No association between SDF1α level and EPC function measured by EPC-CFU was detected in this study.

### Association of *SDF1* genotyping, plasma SDF1α level and circulating EPC number

Allele and genotype frequencies of the *SDF1* and *CXCR4* SNPs examined in this study are shown in **Table 2** **.** The genotype distributions were consistent with Hardy-Weinberg equilibrium.

**Table 2:** Genotype and allele frequencies of *SDF1* and *CXCR4* SNPs studied

Gene	SNP	Genotype	N (%)	Allele	Frequency
*SDF1*	rs2297630	GG	463 (60.05)	G	0.78
		AG	271 (35.15)	A	0.22
		AA	37 (4.80)		
	rs266085	GG	307 (42.05)	G	0.63
		AG	311 (42.60)	A	0.37
		AA	112 (15.34)		
		AG	351 (46.61)	A	0.37
		AA	104 (13.81)		
	rs1801157	GG	445 (62.94)	G	0.79
		AG	231 (32.67)	A	0.21
		AA	31 (4.38)		
	rs1413519	GG	462 (63.20)	G	0.79
		CG	238 (32.56)	C	0.21
		CC	31 (4.24)		
*CXCR4*	rs16832740	AA	447 (62.34)	A	0.79
		AG	237 (33.05)	G	0.21
		GG	33 (4.60)		
	rs12691874	AA	189 (26.21)	A	0.52
		AG	371 (51.46)	G	0.48
		GG	161 (22.33)		

**Figure 2:**
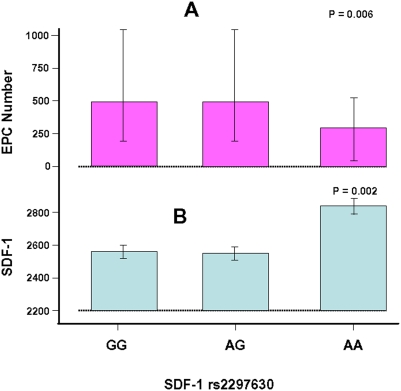
Associations of the SDF1 rs2297630 SNP (2000) with EPC number (median and IQR, A) and blood SDF1α level (arithmetic means and SD, B) assessed in the 2005 evaluation of the Bruneck Study.

The *SDF1* rs2297630 SNP was associated with plasma SDF1α level (p = 0.002) and circulating EPC number (p = 0.006), with the A/A genotype associating with higher SDF1α level and lower EPC number (**Figure 2** **&** **Table 3**). These associations remained significant after Bonferroni correction for the number of SNPs tested (Bonferroni corrected p = 0.014 and p = 0.042, respectively). The associations also remained significant after adjusting for cardiovascular risk factors and the factors that were associated with SDF1α levels, including LDL and HDL cholesterol concentrations, smoking, hypertension, diabetes, alcohol consumption, and levels of hsCRP, MMP9, cystatin C, fibrinogen and homocystein (**Table 3**).

**Table 3:** Plasma SDF1α levels and circulating EPC numbers according to *SDF1* and *CXCR4* SNP genotypes.

SNP	Genotype	SDF1α level	P value	EPC number	P value
SDF1 rs2297630	GG	2562 (2515–2609)	0.002	382 (336–434)	0.006
	AG	2524 (2461–2586)	(0.002)	409 (344–486)	(0.015)
	AA	2863 (2684–3043)		175 (107–286)	
SDF1 rs266085	GG	2572 (2512–2631)	0.661	354 (300–417)	0.793
	AG	2539 (2480–2597)	(0.328)	383 (326–449)	(0.866)
	AA	2529 (2431–2626)		362 (277–473)	
SDF1 rs266087	GG	2570 (2510–2630)	0.931	355 (302–418)	0.702
	AG	2574 (2519–2630)	(0.742)	391 (336–455)	(0.803)
	AA	2552 (2450–2654)		378 (286–499)	
SDF1 rs1801157	GG	2592 (2544–2641)	0.260	370 (323–425)	0.998
	AG	2547 (2483–2612)	(0.208)	368 (307–441)	(0.931)
	AA	2467 (2295–2638)		369 (227–598)	
SDF1 rs1413519	GG	2562 (2513–2611)	0.225	338 (295–386)	0.300
	CG	2588 (2522–2655)	(0.222)	401 (335–482)	(0.229)
	CC	2725 (2541–2909)		397 (240–658)	
CXCR4 rs16832740	AA	2557 (2509–2606)	0.424	374 (328–426)	0.878
	AG	2556 (2487–2624)	(0.747)	395 (329–475)	(0.783)
	GG	2863 (2501–2854)		370 (230–595)	
CXCR4 rs12691874	AA	2514 (2437–2591)	0.306	414 (336–512)	0.272
	AG	2588 (2534–2641)	(0.600)	362 (313–419)	(0.169)
	GG	2571 (2488–2654)		322 (256–404)	

SDF-1 levels and EPC numbers (per 1 ml blood) are age- and sex-adjusted arithmetic (95%CI) and geometric means (95%CI), respectively. P values are from general linear models adjusted for age and sex and in brackets from models additionally adjusted for canditate vascular risk factors and determinants of SDF-1α levels (LDL and HDL cholesterol [mg/dL], smoking [0,1], hypertension [0,1], diabetes [0,1], alcohol consumption [gram/day], homocystein [µmol/L], hsCRP [mg/L], MMP9 [ng/mL], cystatin C [mg/L], fibrinogen [mg/dL]).

Plasma SDF1α level and circulating EPC number was not associated with the other *SDF1* or *CXCR4* SNPs tested. No association was observed between any of the SNPs tested and EPC-CFU.

## Discussion

The population-based, longitudinal design of the Bruneck study allowed us to investigate the relationships between SDF1α levels at two different time points (2000 and 2005) and the relationships of the SDF1α levels at these different time points with circulating EPC numbers. A key finding from our study is the high degree of correlation the two SDF1α measurements in blood samples taken in 2000 and 2005 respectively, suggesting that although SDF1α levels are correlated with cardiovascular risk factors, the levels in an individual are relatively consistent over time. In addition, the study revealed an association between SDF1α level in 2000 and EPC number in 2005, suggesting a long-term predictive value of SDF1α level for EPC number. Another important, novel finding of this study is that there is an association between variation in the *SDF1* gene, SDF1α level and EPC number, indicating that the inter-individual variability in SDF1α level and EPC number is influenced by variation in the *SDF1* gene.

SDF1 is a member of the chemokine CXC subfamily originally isolated from murine bone marrow stromal cells and is expressed also by stromal cells of various tissues[33]. Animal studies have shown that increasing SDF expression by adenovirus gene transfer can induce EPC mobilization from bone marrow into the peripheral blood, thereby increasing circulating EPC numbers[21]–[23], enhancing angiogenesis and blood perfusion in ischemic tissues[25]. These studies have led to the hypothesis that SDF1 plays an important role in EPC mobilization, differentiation and homing. However, data from our study showed that SDF-1α levels were inversely, rather than positively, associated with circulating EPC numbers. There are several possible explanations. Almost all of the exiting data that suggest that SDF1α can induce circulating EPCs are from studies in mouse, and the situations in humans may be different and more complex. Indeed, a number of studies including the present study have shown that EPC numbers in humans are related to a number of factors including age, gender, smoking, lipid levels, hypertension, diabetes, etc. Secondly, it is possible that the relationship of SDF1 with EPC mobilization, differentiation and homing in the acute phase is different from that in the normal situation. Studies of mouse ischemia models showed that the number of c-Kit^+^ cells in peripheral blood was lower but the level of SDF1α was much higher at 14 days after ischemia, as compared with control mice[26], suggesting that EPCs are mobilized into peripheral blood from bone marrow after the onset of ischemia, but at a later stage, the numbers of mobilized EPC in peripheral blood decrease due to their homing to the ischemic site. Thirdly, because SDF1α functions as a chemoattractant for stem/progenitor cells, the SDF1α gradient between bone marrow, peripheral blood and injured tissue/sites is crucial for stem/progenitor cell mobilization, differentiation and homing[26], [34]–[36]. It has been reported that stem/progenitor cells in bone marrow and peripheral blood are mobilized and homed to the ischemic or injured tissue due to high SDF1α levels in the ischemic or injured tissue[26] and lower levels of SDF-1α in the bone marrow[37]. Measurement of plasma SDF1α level does not represent the gradient mention above, and therefore, the inverse relationship between plasma SDF1α level and circulating EPC number should not be interpreted as in conflict with the notion that SDF1 plays an important role in EPC mobilization, differentiation and homing.

A recent study showed that there is significant correlation in the number of circulating EPCs between parents and their offsprings, leading to the hypothesis that EPC number is, at least in part, genetically regulated[29]. The results of our study support this hypothesis and indicate that such a genetic influence is likely in part due to variation in the *SDF1* gene. In particular, we found that SDF1α level and circulating EPC number are associated with the *SDF1* gene rs2297630 SNP in the Bruneck study cohort. A potential interpretation for this novel finding is that variation in the SDF1 gene can influence EPC number via an effect on the level of SDF1α. The rs2297630 SNP is located in intron 3 of the *SDF1* gene. It is possible that the association of this SNP with SDF1α level and circulating EPC number has arisen from a direct functional effect of this SNP on SDF1 expression or mRNA splicing. Alternatively, the rs2297630 SNP might be a functionally neutral marker that is in linkage disequilibrium with a functional polymorphism located elsewhere at the *SDF1* locus.

Two other *SDF1* SNPs, rs266085 and rs1801157, have been reported to be associated with SDF1 levels[27], [28] and the latter SNP has also been reported to be associated with CD34^+^ cell mobilization[30]. Some, but not all, studies have suggested that the rs1801157 SNP is associated with HIV infection and AIDS[38]–[40] and there is also evidence suggesting that this SNP is associated with acute myeloid leukaemia[41], chronic myelogenous leukaemia[42], colorectal cancer[43], sporadic breast cancer[44], carotid artery stenosis[45], type I diabetes[46] and systemic lupus erythematosus[47]. However, in vitro experiments show that this SNP (rs1801157) does not have a direct functional effect on SDF1 expression[48], raising the possibility that it may be a marker for a functional SNP at the *SDF1* locus due to linkage disequilibrium. This possibility is consistent with the fact that the SDF1 gene is transcribed into two isoforms, SDF1α and SDF1β, and that the rs1801157 SNP is located in the 3′ untranslated region of the SDF1β transcript but not in the SDF1α transcript[49], [50]. In the Bruneck study cohort, we found that the rs2297630 SNP, but not the rs266085 or rs1801157 SNP, was associated with SDF1α level and EPC number. Taken together, the studies mentioned above and the present study suggest that variation in SDF1 gene has an influence on SDF1 levels. The populations examined in these different studies may have different genetic structure and linkage disequilibrium patterns, which may explain why different SDF1 SNPs were associated with SDF1 levels in the different studies.

Some particular attentions should be drawn regarding our findings. Firstly, the consideration of the nature of EPC is important, since there is no a unique criteria for identification of EPC, yet. Immense attention regarding the definition of EPC has recently been drawn due to the fact that the characterization and function of EPC isolated with different methodologies were quite different from each other[51]. Myeloid PBMNC derived spindle like EPC, but not circulating CD34+ cells was used to quantify the circulating EPC number in our study. Although a positive relationship between them was reported in the literature, the number of cultured EPC may not always reflect the number of circulating CD34+ and/or KDR+ EPC. Secondly, many diseases have influence on the role of EPC number, such as diabetes[10], [11], smoking[12], rheumatoid arthritis[13] or chronic renal failure[14], among them much reduced EPC number have been reported. Extensive adjustments for cardiovascular risk factors and diseases have been conducted in our study to exclude any potential influence on our findings. Finally, no relationship or association between EPC-CFU and plasma SDF-1α levels and SDF-1 gene SNPs were observed in our study, which indicating that neither plasma SDF-1α levels nor SDF-1 SNP could affect the proliferation and migration of EPC reflected by their ability to form EPC colonies in the culture.

In summary, our data indicate that although the SDF1 level and EPC number may both increase in response to acute ischaemic events, the relationship between blood SDF1 level and EPC number is complex and they are inversely correlated in the normal situation as observed in the Bruneck study cohort which was recruited from the general population. Our data also indicate that circulating EPC number is influenced by variation in the SDF1 gene likely via an effect of the genetic variation on SDF1α expression. These findings help understand the mechanisms underlying the inter-individual variability in EPC number which has an implication in the pathogenesis of atherosclerosis and other cardiovascular diseases.
